# Design of Lidar Receiving Optical System with Large FoV and High Concentration of Light to Resist Background Light Interference

**DOI:** 10.3390/mi15060712

**Published:** 2024-05-28

**Authors:** Qingyan Li, Shuo Wang, Jiajie Wu, Feiyue Chen, Han Gao, Hai Gong

**Affiliations:** 1 School of Control Science and Engineering, Zhejiang University, Hangzhou 310027, China; liqy@hizju.org (Q.L.); wujiajie1270@163.com (J.W.); 2 Intelligent Optical Sensing and Control Center, Huzhou Institute of Zhejiang University, Huzhou 313000, China; asxx40229@163.com (S.W.); chenfeiyue1231@163.com (F.C.); pursue_225@163.com (H.G.); 3 Key Laboratory of Intelligent Photoelectric High-End Equipment in Huzhou, Huzhou 313000, China; 4 School of Advanced Technology, Xi’an Jiaotong-Liverpool University, Suzhou 215123, China

**Keywords:** image telecentric lens, microlens array, anti-background light interference, large field of view, high light concentration

## Abstract

Lidar has the advantages of high accuracy, high resolution, and is not affected by sunlight. It has been widely used in many fields, such as autonomous driving, remote sensing detection, and intelligent robots. However, the current lidar detection system belongs to weak signal detection and generally uses avalanche photoelectric detector units as detectors. Limited by the current technology, the photosensitive surface is small, the receiving field of view is limited, and it is easy to cause false alarms due to background light. This paper proposes a method based on a combination of image-side telecentric lenses, microlens arrays, and interference filters. The small-area element detector achieves the high-concentration reception of echo beams in a large field of view while overcoming the interference of ambient background light. The image-side telecentric lens realizes that the center lines of the echo beams at different angles are parallel to the central axis, and the focus points converge on the same focal plane. The microlens array collimates the converged light beams one by one into parallel light beams. Finally, a high-quality aspherical focusing lens is used to focus the light on the small-area element detector to achieve high-concentration light reception over a large field of view. The system achieves a receiving field of view greater than 40° for a photosensitive surface detector with a diameter of 75 μm and is resistant to background light interference.

## 1. Introduction

Autonomous driving technology is developing rapidly and aims to provide a safe, convenient, and comfortable driving experience. Environment perception, positioning, and mapping capabilities are critical to achieving a high level of autonomous driving. lidar has the characteristics of high accuracy, high resolution, and anti-interference, and is one of the indispensable sensors for autonomous driving [1,2,3,4,5]. Compared with microwave radar, lidar has higher accuracy and a longer detection range. Compared with cameras, lidar has distance information and is not affected by lighting. Therefore, lidar has become a research hotspot around the world, and lidar technology has been widely used in many fields.

At present, many design methods have been proposed to expand the imaging receiving field of view (FoV) of traditional receiving optical systems, such as wide-angle lenses and fisheye lenses. However, for imaging lidar, the signal detection is weak signal detection. Generally, an avalanche photodetector unit is used as the detector. Limited by the current technology, the photosensitive surface is small, the receiving FoV is limited, and false alarm signals are easily generated due to background light [6,7,8].

In addition, the pulse width of the emitted laser is only a few ns, so a very fast response speed and response bandwidth are required for the subsequent signal readout circuit, which is another great challenge [9,10,11]. On the other hand, the single-element gaze receiving system has a simple structure, which can greatly simplify the design of the system and achieve miniaturization. Further, it can improve the overall stability and practicality of imaging lidar and expand its application scope. Therefore, how the lidar system overcomes the small receiving FoV of the unit detector and overcomes the background light interference is currently a technical difficulty. To solve this problem, the traditional method is to add auxiliary optical components, such as field lenses [12], light cones [13], or immersion lenses [14]. Through the above methods to expand the receiving FoV, the range of the receiving FoV can be appropriately increased while ensuring the focusing ability. However, these solutions have a limited effect on expanding the receiving FoV of small-area element detectors and cannot meet the actual application needs.

In addition, the Harbin Institute of Technology proposed a design scheme to increase the lidar receiving FoV through beam splitting with a hexagonal prism [15]. Two hexagonal prisms are combined. After the echo beam passes through the system, the beam is divided into seven beams, which are matched with seven aspherical lenses for detection. This solution increases the receiving FoV to ±5°. However, the system structure is relatively complex, the volume is large, and there are disadvantages of uneven beam quality after focusing.

Currently, high-concentration light-receiving systems based on non-imaging theory are mainly used in solar energy collection and LED lighting technology. The main concentrator solutions include refractive, reflective, and hybrid concentrator systems. The main focus of the light condensing system is the control of light energy transmission rather than the one-to-one correspondence between objects and images. The standard for evaluating its system performance is light energy utilization. Refractive concentrators are often based on Fresnel lenses to achieve the high-performance convergence of light beams. In 2016, the thermal energy science team of the University of Science and Technology of China designed a point-focusing Fresnel lens condensing system [16]. By combining a Fresnel lens and an optical prism, the effect of condensing light first and then uniforming the light is achieved. At the same time, the area of the condensing lens can be much larger than the area of the photovoltaic cell such that the system achieves a high light concentration ratio. The refractive condensing system has a high degree of design freedom, but the refractive lens is so sensitive to the spectrum that it is prone to dispersion, and the structure of the Fresnel lens is relatively complex.

Hybrid concentrating systems have been widely promoted in recent years due to their compact structure, high concentrating efficiency, and ability to provide dynamic concentrating capabilities. Hybrid concentrating systems typically combine focusing methods such as refraction, reflection, and total internal reflection. In 2010, the University of California proposed a flat-panel light-concentrating system based on a planar microlens array [17]. The light beam is focused on the light guide plate through the microlens array, and the light guide plate serves as a secondary element to guide the light to the photovoltaic cells at the edge. In 2021, a research team from the Hefei University of Technology proposed a compound parabolic eye concentrator system [18]. The compound eye lens has the advantages of a compact structure, large receiving FoV, and small size. In addition, it uses a compound parabolic focusing system for secondary focusing, which overcomes the problem of beam dispersion. The overall system has a large receiving FoV and high light concentration efficiency.

In general, the current method for achieving large-FoV reception with small-area element detectors is mainly to add auxiliary optical components, such as field lenses, light cones, and immersion lenses, and to configure catadioptric lenses or prisms. Adding auxiliary optical components can increase the receiving FoV of the detector to a certain extent, but its effect is limited. Its performance is insufficient for imaging ranges with larger fields of view. On the other hand, the catadioptric lens and prism system demonstrate better performance, but the system requires multiple lenses and prisms to be used in combination, which increases the cost and difficulty of assembly and adjustment. Moreover, the volume is large, making it difficult to miniaturize the system. High-concentration light-receiving systems based on non-imaging theory are mainly used in solar energy collection and lighting technology. The system is larger in size and has a larger photosensitive surface. Therefore, it is very important for MEMS imaging lidar to use small-area element detectors to achieve a large FoV to receive echo signals while ensuring high light concentration efficiency.

To this end, this article proposes a combination of an image-side telecentric lens and a microlens array to correct the echo laser beams in different fields of view into parallel beams. Then, the beams are focused on the small-area element detector through a high-performance aspheric focusing lens, thereby realizing the large-FoV reception of the small-area element detector. In addition, the above system avoids the influence of different incident angles of interference filters, thereby overcoming background light interference. At present, the application of lidar has put forward higher requirements for related technologies, requiring the system to be free from background light interference, with higher detection efficiency, longer detection distance, and a larger field of view. The design of this system can overcome the problems of a limited receiving field of view, background light interference, and receiving efficiency in the current lidar systems. This design can further improve the performance and practicality of lidar systems.

## 2. Theoretical Analysis

### 2.1. Effect of Background Light Radiation on Echo Signals

During the signal receiving process of the receiving optical system, in addition to the echo signal of the target, other photoelectric signals entering the receiving system are a kind of interference. The target’s echo signal and various background radiation noises will inevitably be mixed together. The larger the FoV of the receiving system, the more noise will enter the system, which will have a great impact on the detection of long-distance weak echo signals in the system. The noise entering the system can be divided into background light radiation noise, detector noise, electronic component thermal noise, etc. In the system, the noise can be reduced to negligible over the echo signal through the optimized design of the detector and electronic components. So, the background light noise is regarded as the main source of noise in this article.

**Figure 1:**
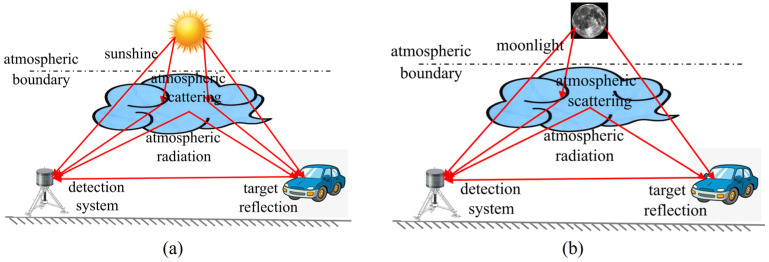
Effect of background light radiation noise. (**a**) Noise sources during the day; (**b**) noise sources at night.

Background noise refers to the flicker noise generated by the energy of background natural radiation (including sunlight reflection) passing through the detector, which is fundamentally derived from the thermal radiation of objects. The background radiation noise entering the detector can be divided into direct radiation noise and scattering noise. During the day, the direct radiation noise mainly includes solar radiation and the detection target’s own radiation. The scattering noise includes primary scattering noise such as solar radiation reflected by the detection target and solar radiation scattered by atmospheric molecules, as well as the secondary scattering noise caused by the atmospheric thermal radiation reflected by the target, as shown in Figure 1a. During the night, the noise signal does not have direct radiation energy from sunlight. The direct radiation noise is only the thermal radiation of atmospheric molecules in the sky and the detection target’s own radiation, while the scattered noise is the moonlight and the target’s reflection of atmospheric radiation and moonlight, as shown in Figure 1b.

According to the object radiation theory, as long as the temperature of an object is greater than absolute 0 K, thermal radiation will be generated on its surface. The spectral radiation of a black body conforms to Planck’s theorem, and the irradiance can be expressed as

(1) $$L(\lambda ,T)=\frac{2hc^{2}}{\lambda^{5}}\cdot \frac{1}{e^{\frac{hc}{\lambda kT}}-1}$$

where *λ* is the radiation wavelength, *h* ($h=6.6260755\times 10^{-34}\;J\cdot s$) is Planck’s constant, *c* is the speed of light in vacuum, *k* ($k=1.380658\times 10^{-23}\;J/K$) is Boltzmann’s constant, and *T* is the absolute temperature of the object (K).

**Figure 2:**
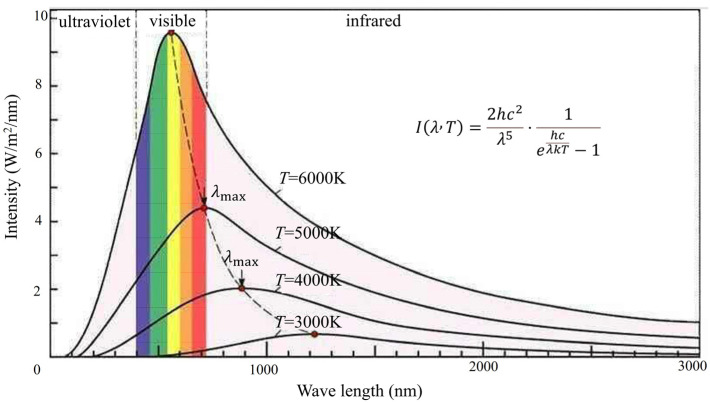
Radiation curves of blackbodies at different temperatures at different wavelengths [19].

Figure 2 shows the radiation curves of blackbodies at different temperatures at different wavelengths [19]. Although the detection target is different from black body radiation, its spectral energy radiation characteristics are similar to those of a black body, so this theory can be used for approximate calculations. It can be seen from the radiation curve that the blackbody radiation intensity has a radiation peak wavelength. The peak wavelength changes according to the temperature of the object. As the blackbody temperature decreases, the radiation peak wavelength moves toward the long wave direction.

**Figure 3:**
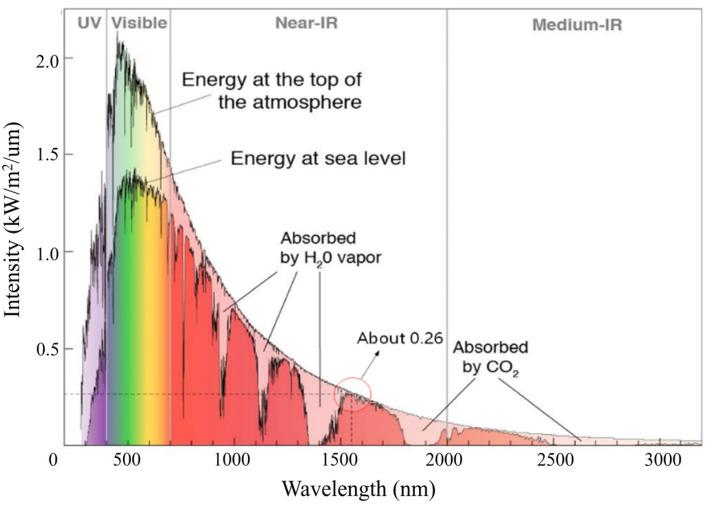
Spectral irradiance of the Sun at sea level [20].

Generally speaking, the Sun can be regarded as an absolute black body with a temperature of 6000 K. The average temperature of Earth’s surface is 300 K. The radiation intensity curve of sunlight in Earth’s atmosphere and sea level is shown in Figure 3 [20].

The amount of solar radiation entering the sea level in the 1550 nm band is smaller than in other bands, so it has an absolute advantage in overcoming background radiation. The solar radiation intensity in the 1550 nm band is $I_{sun}(1550)=260{\;W/m}^{2}/\mu m$. If the detection system is directly facing the sky, the solar radiation noise directly entering the receiving system is

(2) $$P_{n-sun}=\eta_{r}A_{r}I_{sun}(1550)B_{\Delta \lambda }$$

where $B_{\Delta \lambda }$ is the filter bandwidth of the optical system, $\eta_{r}$ is the transmittance of the receiving optical system, and $A_{r}$ is the area of the receiving optical system.

When the imaging radar system is not directly facing the sky, the solar noise received by the system detector mainly comes from the reflection of objects. The energy of the sunlight reflected by the detection target entering the detector is

(3) $$P_{n-sun}=\eta_{r}\frac{\pi }{4}{\theta_{optic}}^{2}A_{r}\rho_{T}I_{sun}(1550)B_{\Delta \lambda }\cos\theta_{\lambda }$$

where $\theta_{optic}$ is the receiving aperture angle of the optical system, $\theta_{\lambda }$ is the angle between the receiving optical system and the center of the reflected beam, and $\rho_{T}$ is the average reflectivity of the detection target surface.

**Figure 4:**
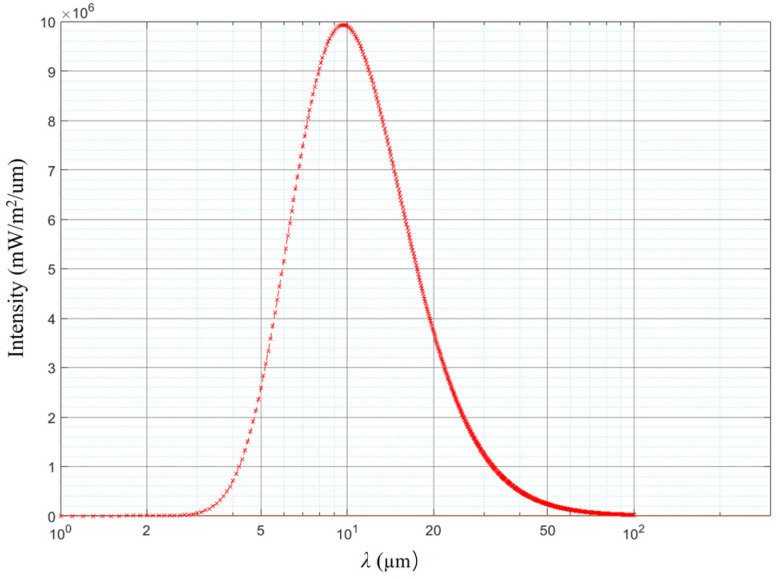
Radiation curves of a target object with a temperature of 300 K in different bands.

According to Planck’s theorem, the thermal radiation energy of a target object at 300 K on the ground in different wavebands can be obtained. As shown in Figure 4, the optical radiation intensity at 1550 nm can be obtained as $I_{t}(1550)=4.7\times 10^{-4}{\;W/m}^{2}/\mu m$.

Then, the thermal radiation noise of the detection target’s own thermal radiation entering the receiving system is

(4) $$P_{n-sun}=\eta_{r}A_{r}I_{sun}(1550)B_{\Delta \lambda }$$

For scattering background noise, the optical radiance of atmospheric scattering of sunlight at the 1550 nm band is approximately $I_{atm}(1550)=5{\;W/m}^{2}/\mu m$. Therefore, during the day, the noise caused by the scattering of sunlight by the atmosphere entering the receiving system is

(5) $$P_{n-sun}=\eta_{r}\frac{\pi }{4}{\theta_{optic}}^{2}A_{r}\rho_{T}I_{sun}(1550)B_{\Delta \lambda }\cos\theta_{\lambda }$$

In the same way, during the night, the energy of the moonlight reflected by the target entering the system for detection is

(6) $$P_{n-sun}=\eta_{r}A_{r}I_{sun}(1550)B_{\Delta \lambda }$$

**Figure 5:**
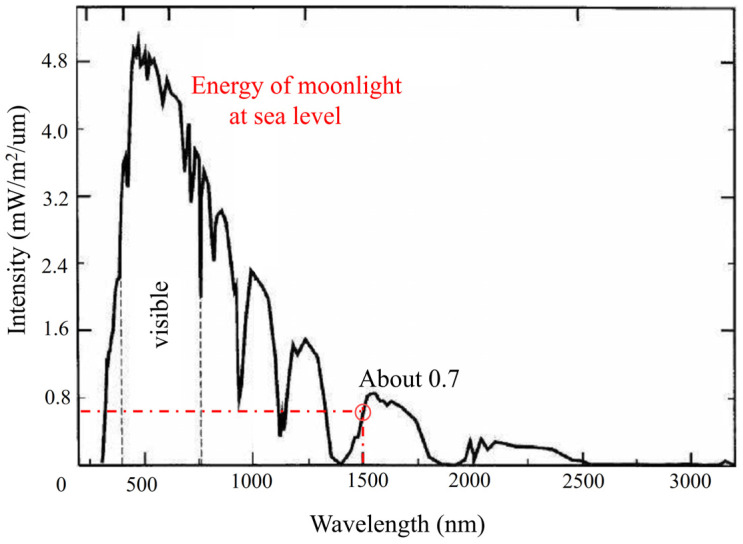
Spectral irradiance of the Moon at sea level.

Figure 5 shows the spectral irradiance of the Moon at sea level. From Figure 4, it can be seen that the spectral irradiance at 1550 nm is $L_{moon}(1550)=7\times 10^{4}\;{W/m}^{2}/\mu m$.

Therefore, the radiated noise power entering the detector during the day and night can be obtained as

(7) $$P_{n-sun}=\eta_{r}\frac{\pi }{4}{\theta_{optic}}^{2}A_{r}\rho_{T}I_{sun}(1550)B_{\Delta \lambda }\cos\theta_{\lambda }$$

(8) $$P_{n-sun}=\eta_{r}A_{r}I_{sun}(1550)B_{\Delta \lambda }$$

Table 1 provides the initial parameter values of the system and the calculation results of the relevant noise power. Judging from the calculation results in the table, the noise power at night is 5 orders of magnitude lower than the noise power during the day. Sunlight noise has a greater impact on detection than other noise entering the detector. Therefore, imaging lidar should try to avoid direct sunlight and install narrow-band filters during operation. If there is a demand to detect targets in the sky, coherent detection needs to be used to overcome solar noise.

**Figure 6:**
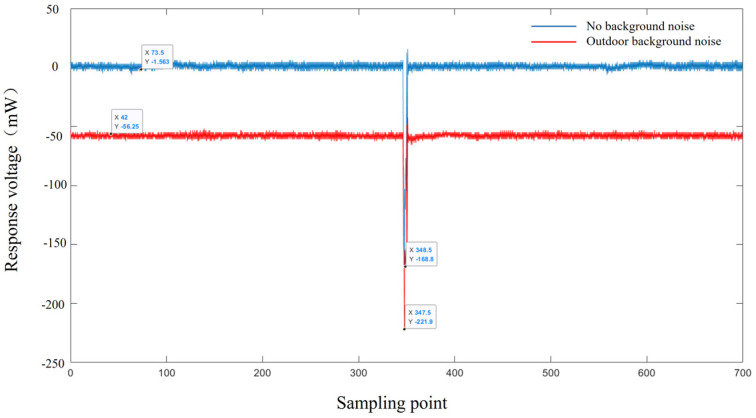
Results of outdoor background light test.

The background noise test is shown in Figure 6. It can be observed that sunlight background light has a significant impact on the detection of weak signals. Therefore, imaging lidar should try to avoid direct sunlight and install narrow-band filters during operation.

**Figure 7:**
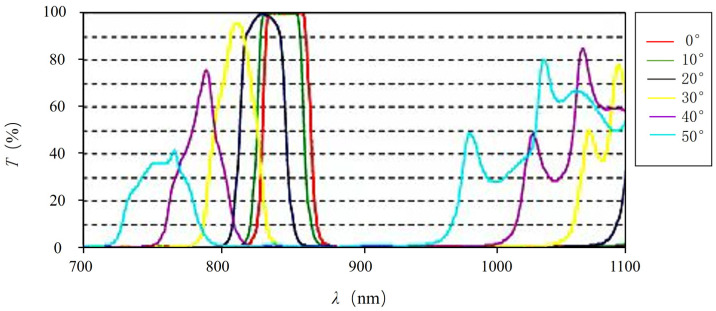
Filtering performance of narrow-band filters for laser beams of different wavelengths at different incident angles.

Absorption type and interference type are two main types of narrowband filters. Absorption filters are mainly based on colored glass, taking advantage of its ability to absorb specific wavelengths of light. The advantages of absorption filters include good stability, uniformity, good beam quality, and low manufacturing cost. However, the disadvantage is that the passband is relatively large, usually greater than 30 nm, and the detection performance for weak signals is poor. On the other hand, interference filters are optical film layers of specific thickness that are applied to the surface of glass by vacuum coating. A piece of glass is composed of multiple layers of thin films based on the principle of interference to allow light waves in a specific spectral range to pass through. It is characterized by a narrow passband and better filtering performance. However, based on the principle of interference, different incident angles will lead to different optical path differences, so interference filters are very sensitive to the incident angle. Figure 7 shows the filtering performance of the narrow-band filter for laser beams of different wavelengths at different incident angles. As the incident angle increases, the center wavelength shifts and the filtering performance decreases. Therefore, it is also difficult for scanning imaging lidar to overcome background light interference in different fields of view. When designing the receiving optical system, the influence of background light interference needs to be considered, and the problem of center wavelength shift caused by differences in incident angles needs to be overcome.

### 2.2. Design of Optical System for Receiving Echo Signals with Large FoV and High Concentration

**Figure 8:**
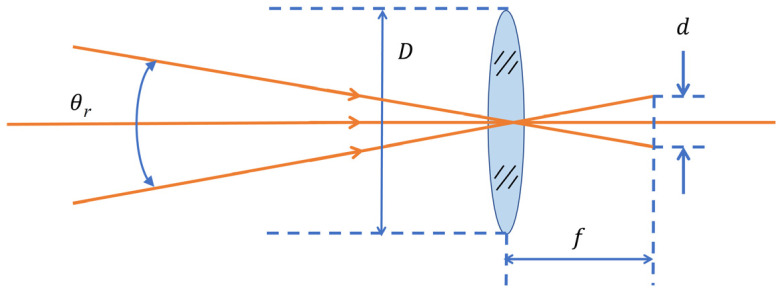
The relationship between detector diameter and receiving FoV.

In the imaging system, the detector is located at the focal plane of the receiving lens, which can achieve maximum efficiency of receiving the light beam. The size of the photosensitive surface of the detector directly determines the size of the receiving FoV of the system. The relationship between the two is shown in Figure 8.

The relationship between the receiving FoV $\theta_{r}$, the focal length *f* of the receiving lens, and the detector diameter *d* can be expressed as

(9) $$\theta_{r}=2\arctan\frac{d}{2f}\approx \frac{d}{f}$$

If the system wants to achieve large-FoV reception, the detector diameter needs to be increased accordingly. For detectors with large photosensitive surfaces, the larger the photosensitive surface, the stronger the speckle effect, which will reduce the signal-to-noise ratio of the system. In addition, the response bandwidth is also smaller, and, the larger the photosensitive surface, the higher the cost. Array and area array high-performance APD detectors are difficult and costly to manufacture, and the response bandwidth will be reduced. In addition, the subsequent transimpedance amplification circuit and signal reading circuit are also very difficult.

**Figure 9:**
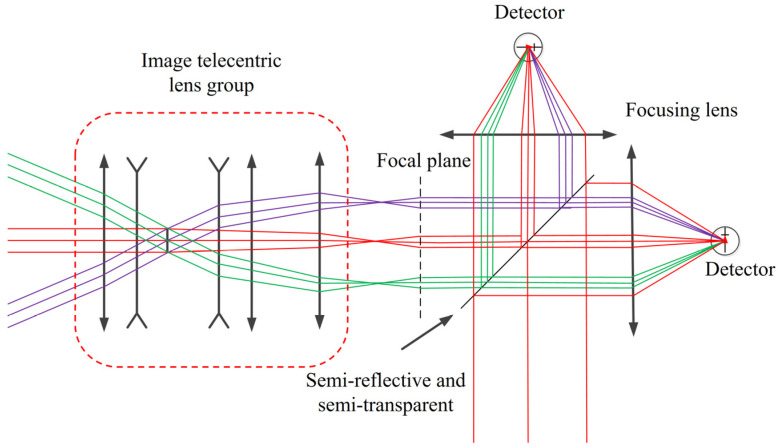
Optical path diagram of large-FoV and high-concentration light-receiving system.

Taking into account the problem of small-area element detectors and the background light interference of different fields of view, this project proposes an optoelectronic system that can realize large-FoV signal reception by small-area element detectors, as shown in Figure 9. The system uses a combination of an image telecentric system and a microlens array to correct the spatial lattice beams from different fields of view to achieve parallel light output and overcome the impact of the incident angle on the interference filter. Moreover, the system realizes that, after the echo signals of different fields of view pass through the image-side telecentric objective lens, their chief rays are parallel to the optical axis. The concentrated light spot falls on the focal plane, and the spatial lattice formed corresponds to the microlens array unit lens one-to-one. It is then converged to a small-area element detector through a high-performance aspheric focusing lens to achieve large-FoV detection. In addition, this solution is highly scalable and can achieve high-performance coherent detection. The combination of the image telecentric system and the microlens array ensures the spatial coherence of the echo beam. The corrected echo beam achieves an efficient coherence with the local oscillator light through the half mirror.

The focal lengths of the two focusing lenses behind the dichroic prism are the same. By adding a dichroic prism for light splitting, the system can use two detectors with the same performance for reception to achieve double-balanced detection. The signals received by the two detectors are processed through signal processing, which can overcome the noise caused by its own electronic components, and the system can detect weaker signals.

## 3. Optical Design and Parametric Analysis

**Figure 10:**
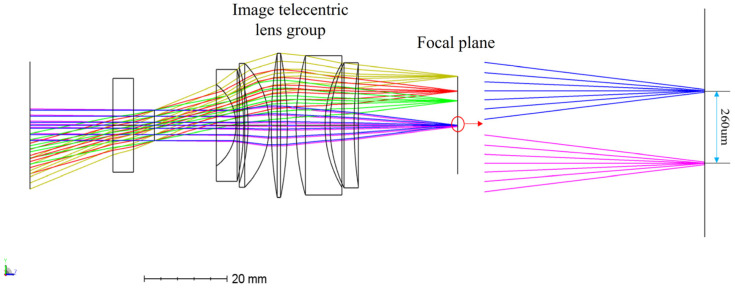
Image-side telecentric lens structure diagram.

**Figure 11:**
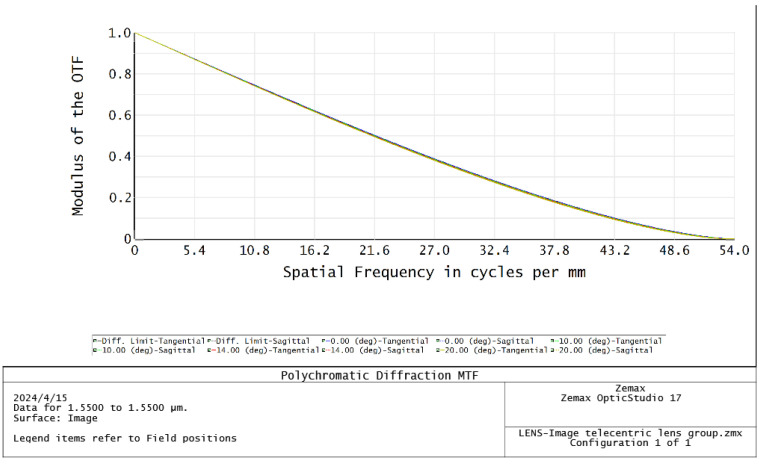
Image-side telecentric lens MTF function curve diagram.

Large-FoV telecentric lenses are designed first. The chief ray of the echo beam after passing through the image-side telecentric lens is parallel to the optical axis, and the beam image height is proportional to the receiving FoV. Through ZEMAX (2017) software design and optimization, a five-piece large-FoV telecentric lens system is finally obtained. The specific structure and optical path are shown in Figure 10. When the FoV of the receiving beams differs by 0.5°, the distance between the converged beams is 260 μm. The MTF function image of the telecentric lens is shown in Figure 11. The MTF function curve is smooth and close to the diffraction limit, which meets the design requirements. The image telecentric lens is composed of five spherical lenses, with a maximum receiving field of view of 40°. The parameter values of the image-side telecentric lens are shown in Table 2.

**Figure 12:**
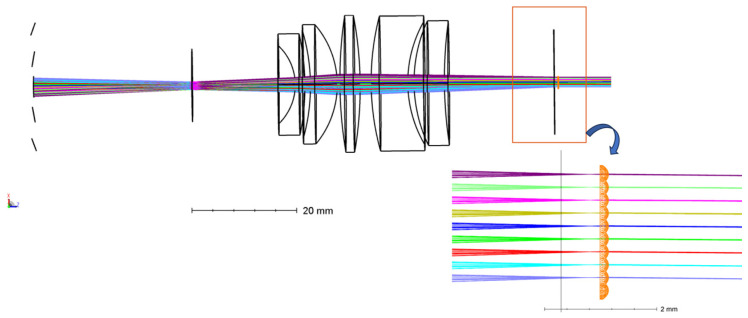
The optical path diagram of the echo beam in the paraxial region passing through the combination of the image-side telecentric lens and the microlens array.

**Figure 13:**
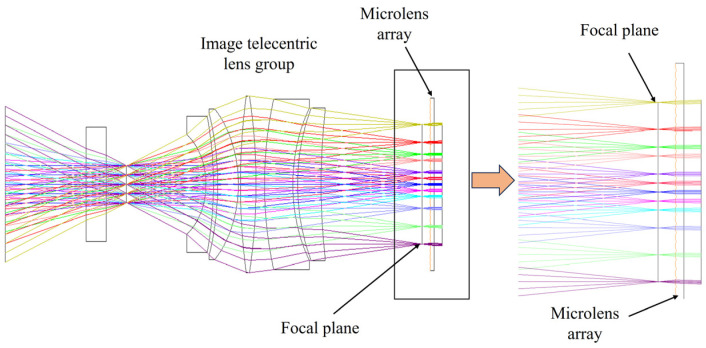
The optical path diagram of the echo beam of the entire FoV passing through the combination of the image-side telecentric lens and the microlens array.

A microlens array is added after the image-side telecentric lens for beam correction. The parameters of the microlens array are determined by comprehensively considering the position of the echo beams of different fields of view of the image-side telecentric lens on the image-side focal plane and the spatial resolution of the system. The echo beam is corrected into a parallel beam after passing through the microlens array. Figure 12 is the optical path diagram of echo beams at different angles in the paraxial region passing through the image-side telecentric lens and microlens array. The blue beam is the central FoV echo beam. Different colors represent beams with different receiving angles. The receiving angle difference between two adjacent beams is 0.5°. The overall structure and optical path of the entire receiving FoV are shown in Figure 13. The echo beams of different receiving fields of view can be well-collimated.

**Figure 14:**
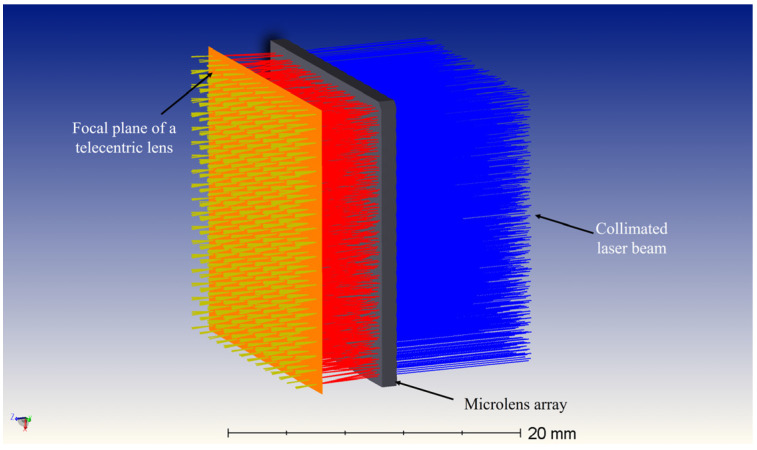
Microlens array structure diagram.

The structure of the microlens array is shown in Figure 14. The echo beam in the full FoV is collimated into a parallel beam after passing through the microlens array.

**Figure 15:**
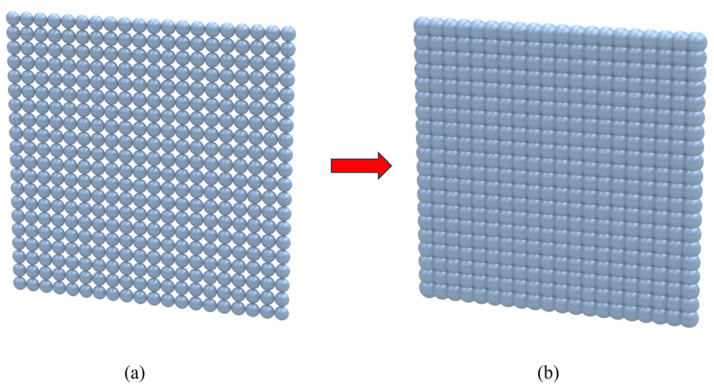
Microlens array structure diagram: (**a**) traditional microlens array structure; (**b**) the improved microlens array structure in this article.

**Figure 16:**
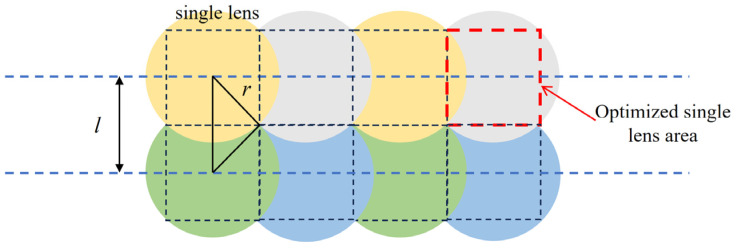
Optimization scheme of microlens array.

In addition, the traditional microlens array arrangement is shown in Figure 15a. The spherical single lenses are arranged regularly. However, the disadvantage of this design is that there is a gap in the middle. The gap position is replaced by a glass plate, so this part does not play a role in beam correction. Therefore, this article optimizes the above structure, and the optimized structure is shown in Figure 16. Finally, in order to ensure that the center distance of the two microlens units is still 260 μm, the original single lens is enlarged by $\sqrt{2}$ times. The edge portions of adjacent lenses are overlapped until there is no gap. Then, only the central part of each single lens is retained for regular arrangement. The optimized microlens array structure is shown in Figure 15b.

**Figure 17:**
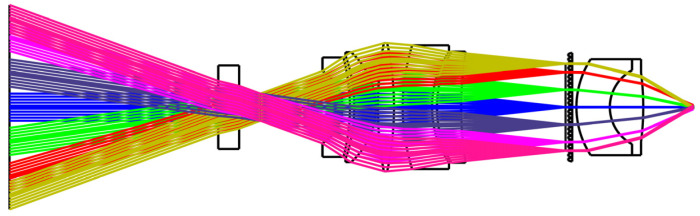
Overall optical path diagram of the large-FoV receiving optical system.

From the simulation results, the light beams in different fields of view all achieve parallel light emission. By adding an interference filter and a double cemented focusing lens at the rear, the system can overcome the influence of background light radiation in different fields of view and at the same time achieve efficient reception of signal light by small-area detectors. The system structure is shown in Figure 17.

The echo beams from different fields of view converge to the rear focal plane after passing through the image-side telecentric lens. The beam is then corrected into a parallel beam through a microlens array. The optical system also has the same effect on background light noise. Therefore, using an interference filter afterwards can effectively filter out the interference of background light. At the same time, it also overcomes the influence of different incident angles on the performance of the interference filter.

## 4. Discussion

This article uses a combination of image-side telecentric lenses and microlens arrays to correct the echo laser beams in different fields of view into parallel beams. Then, it is focused on the small-area element detector through a high-performance aspheric focusing lens, thereby realizing the large field of view reception of the small-area element detector. In addition, the above system avoids the influence of different incident angles of interference filters, thereby overcoming background light interference. This design can further improve the performance and practicality of lidar systems.

On the other hand, due to the long detection distance, the laser attenuates greatly during atmospheric transmission. For vehicle-mounted lidar, human eye safety must also be considered. The laser energy cannot be increased indefinitely, so the echo signal received by the system is very weak. For some harsh application scenarios, direct detection cannot meet the needs, and the system must use coherent detection, which has strict restrictions on the receiving optical system. In order to obtain effective optical interference on the photosensitive surface of the detector, optical heterodyne detection requires that the local oscillator light and the incident signal light have the same polarization direction and the energy flow vector remains consistent, that is, the angular collimation in space is maintained (alignment and coaxial).

It is also required that the wave fronts of the two light waves must match the curvature. Research shows that, in order to ensure the spatial coherence of the two beams of light, the incident angle θ between the local oscillator light and the signal light satisfies the following:

(10) $$\theta \leq \frac{\lambda }{D}$$

where *λ* is the laser wavelength and D is the diameter of the photosensitive surface of the detector.

In the large-FoV and high-concentration light-receiving optical system designed in this article, the image telecentric lens and the microlens array are combined to collimate the echo beams of different fields of view into parallel beams and then collimate them. Therefore, the incident angle between the echo beam and the reference beam converged on the detector is consistent, which can well meet the interference conditions.

## 5. Conclusions

Taking into account the problems of small-area element detectors and background light interference in different fields of view, this paper proposes an optoelectronic system that can realize large-FoV signal reception by small-area element detectors. When the photosensitive area of the detector is less than 1 mm, the receiving FoV is greater than 40° and can overcome the interference of background light. The system uses a combination of an image telecentric system and a microlens array to correct the spatial lattice beams from different fields of view to achieve parallel light output and overcome the impact of the incident angle on the interference filter. Moreover, by adopting this system, after the echo signals of different fields of view pass through the image-side telecentric objective lens, their chief rays are parallel to the optical axis, the converged light spot falls on the focal plane, and the spatial lattice corresponds to the microlens array unit lens one-to-one. It is then converged to a small-area element detector through a high-performance aspheric focusing lens to achieve large-FoV detection. In addition, the solution is highly scalable and can achieve high-performance coherent detection. The combination of the image telecentric system and the microlens array ensures the spatial coherence of the echo beam. The corrected echo beam achieves efficient coherence with the local oscillator light through the half mirror.

## Figures and Tables

**Table 1:** Background light radiation noise parameters.

Parameter	Value	Parameter	Value
$\eta_{r}$	0.8	$B_{\Delta \lambda }$	10nm
$\theta_{\lambda }$	$90^{\circ }$	$\theta_{optic}$	170mrad
$\rho_{T}$	0.2	$A_{r}$	$6.81\times 10^{-7}{\;m}^{2}$
$L_{sun}(1550)$	$260{\;W/m}^{2}/\mu m$	$L_{t}(1550)$	$4.7\times 10^{-4}{\;W/m}^{2}/\mu m$
$L_{atm}(1550)$	$5{\;W/m}^{2}/\mu m$	$L_{moon}(1550)$	$7\times 10^{-4}{\;W/m}^{2}/\mu m$
$P_{n-sun}$	$3.2\times 10^{-8}\;W$	$P_{n-t}$	$5.784\times 10^{-14}\;W$
$P_{n-atm}$	$6.15\times 10^{-10}\;W$	$P_{n-moon}$	$8.649\times 10^{-14}\;W$
$P_{moon}$	$3.262078\times 10^{-8}\;W$	$P_{night}$	$1.4433\times 10^{-13}\;W$

**Table 2:** The parameter values of the image-side telecentric lens.

Surface: Type	Radius (mm)	Thickness (mm)	Glass	Semi-Diameter (mm)	Mechanical Semi-Diameter (mm)
1: Standard surface	−10.277	1.845	H-QK3L	7.457	9.675
2: Standard surface	−46.106	1.058		9.675	9.675
3: Standard surface	−37.152	5.125	N-SF66	10.179	11.394
4: Standard surface	−17.625	0.500		11.394	11.394
5: Standard surface	83.327	3.869	N-ASF46A	12.939	13.050
6: Standard surface	−73.388	2.099		13.050	13.050
7: Standard surface	50.000	7.000	N-SF6HT	12.884	12.884
8: Standard surface	23.491	1.198		11.756	12.884
9: Standard surface	27.945	5.798	H-ZF7LA	11.911	11.911
10: Standard surface	84.783			11.562	11.911

## Data Availability

The original contributions presented in the study are included in the article, further inquiries can be directed to the corresponding author.
